# Repetitively Mode-Locked Cavity-Enhanced Absorption Spectroscopy (RML-CEAS) for Near-Infrared Gas Sensing

**DOI:** 10.3390/s17122792

**Published:** 2017-12-02

**Authors:** Qixin He, Minhan Lou, Chuantao Zheng, Weilin Ye, Yiding Wang, Frank K. Tittel

**Affiliations:** 1 Department of Electrical and Computer Engineering, Rice University, 6100 Main Street, Houston, TX 77005, USA; qixinhe@rice.edu (Q.H.); ml52@rice.edu (M.L.); wy10@rice.edu (W.Y.); fkt@rice.edu (F.K.T.); 2 State Key Laboratory of Integrated Optoelectronics, College of Electronic Science and Engineering, Jilin University, 2699 Qianjin Street, Changchun 130012, China; ydwang@jlu.edu.cn; 3 College of Engineering, Shantou University, 243 Daxue Road, Shantou 515063, China

**Keywords:** infrared absorption spectroscopy, cavity enhanced absorption spectroscopy, gas sensor

## Abstract

A Pound-Drever-Hall (PDH)-based mode-locked cavity-enhanced sensor system was developed using a distributed feedback diode laser centered at 1.53 µm as the laser source. Laser temperature scanning, bias control of the piezoelectric ceramic transducer (PZT) and proportional-integral-derivative (PID) feedback control of diode laser current were used to repetitively lock the laser modes to the cavity modes. A gas absorption spectrum was obtained by using a series of absorption data from the discrete mode-locked points. The 15 cm-long Fabry-Perot cavity was sealed using an enclosure with an inlet and outlet for gas pumping and a PZT for cavity length tuning. The performance of the sensor system was evaluated by conducting water vapor measurements. A linear relationship was observed between the measured absorption signal amplitude and the H_2_O concentration. A minimum detectable absorption coefficient of 1.5 × 10^–8^ cm^–1^ was achieved with an averaging time of 700 s. This technique can also be used for the detection of other trace gas species by targeting the corresponding gas absorption line.

## 1. Introduction

Laser absorption spectroscopy-based trace-gas sensors are widely used in environmental monitoring, industrial process monitoring, explosive detection and plasma diagnostics, as well as in the life sciences and medical applications [1,2,3]. To achieve a high sensitivity, the multi-pass absorption spectroscopy technique is used, which increases the effective absorption path length while retaining a comparatively small sample volume. Cavity ring-down spectroscopy (CRDS) [4,5] and cavity-enhanced absorption spectroscopy [6,7,8,9,10] (CEAS) are two multi-pass techniques based on optical cavities. In CRDS, the ring-down time representing the decay rate of the light intensity leaking from an optical cavity is calculated from the measured signal and used to determine the gas concentration. Alternatively, in CEAS, the light transmittance at the cavity resonances is measured. This simplifies the signal processing and makes the sensor operation more convenient [11,12,13,14]. There are many types of CEAS that have been reported in the literature, such as optical-feedback cavity-enhanced absorption spectroscopy (OF-CEAS) [4,15,16,17], incoherent broadband cavity-enhanced absorption spectroscopy (IBBCEAS) [18,19], off-axis cavity-enhanced absorption spectroscopy (OA-CEAS) [20,21], and noise-immune cavity-enhanced optical-heterodyne molecular spectroscopy (NICE-OHMS) [22,23]. 

In a continuous mode-locked CEAS setup, the cavity resonant frequency is matched to the laser frequency using a continuous Pound-Drever-Hall (PDH)-based mode-locking technique [24,25]. In this way, the transmitted light intensity from the cavity can be continuously recorded as the laser scans over a targeted gas absorption line. The disadvantages of this technique are that the locked wavelength range is usually limited by the length extension of the piezoelectric ceramic transducer (PZT) and that the sensor system can be perturbed by external disturbances. For the case of the small PZT travel that such a system uses, the laser output wavelength cannot sweep across an entire gas absorption line. Therefore, a traditional mode-locking mechanism cannot be used for this detection system.

As an alternative mode-locking technique, we demonstrated a novel repetitively mode-locked cavity-enhanced absorption spectroscopy (RML-CEAS) technique for near-infrared gas detection. For this technique, the laser wavelength was tuned by temperature scanning to sweep across a gas absorption line. During wavelength scanning, the laser mode was repetitively locked to the cavity mode by feedback control of the laser current using a PDH technique. If the mismatch between the laser mode and the cavity mode is too large to be compensated, the two modes become unlocked and the laser mode waits for relocking to the next cavity mode. Hence a complete gas absorption spectrum can be obtained by using a series of absorption data consisting of discrete mode-locked points. Compared with continuous mode-locking (CML), RML does not require continuous wide wavelength range locking and simplifies the sensor design without deteriorating the sensor performance.

In the reported RML-CEAS sensor an electro-optic modulator (EOM) is used to phase-modulate the incident diode laser light [26,27]. Subsequently, the phase information of the reflected light is detected to generate a frequency error signal. A proportional-integral-derivative (PID) control module generates a feedback control signal to control the laser current. Suitable PID parameters were selected to achieve fast, stable mode locking. PZT bias control was also needed to obtain more mode locking points for a good definition of the gas absorption. When the PZT bias voltage was varied, the locking frequency changed accordingly and more locking points corresponding to different locking positions in the gas absorption curve were obtained by repetitive scanning of the gas absorption line.

## 2. Sensor System Configuration

### 2.1. Sensor Structure

**Figure 1:**
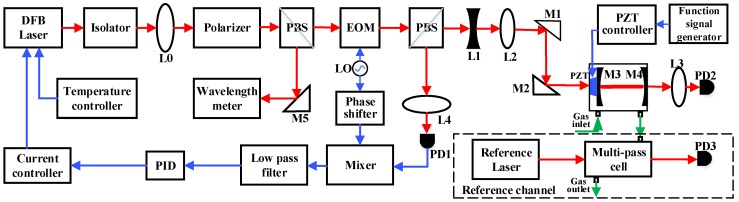
Schematic of the near-infrared repetitively mode-locked cavity-enhanced sensor system. The red lines represent the diode laser beam, and the blue lines represent electrical signals. A reference LDAS (Laser Direct Absorption Spectroscopy)-based sensor system shown by a dashed line including a reference laser, multi-pass gas cell and a photodetector (PD3), were used to measure the H_2_O concentration.

The design of the sensor system is shown in Figure 1. The optical part of the system consists of a distributed feedback (DFB) diode laser, two optical isolators, a polarizer, two beam splitters, an optical cavity as well as several lenses and mirrors. A DFB diode laser (#CQF935/908, JDS Uniphase, Milpitas, CA, USA), emitting at a wavelength of ~1.53 μm) was used as the light source. Because the diode laser is sensitive to optical feedback, two optical isolators (IO-H-1550FC, Thorlabs, Newton, NJ, USA) placed in series were used to attenuate the light reflected from the cavity and the isolation is 29 dB at a wavelength of ~1.53 μm. A collimator (L0) was used to reduce the diode laser beam size and a polarizer was employed to ensure a well-defined polarization of the laser beam. The laser light was then divided into two beams by means of a beam splitter (with a split ratio is 50:50). One beam was directed to a wavelength meter (model 671 Series, Bristol Instruments, Rochester, NY, USA with an accuracy of ±0.0001 nm) in order to measure the diode laser output wavelength. The other beam is directed to the cavity after passing through several optical components. Two lenses (L1, L2) were used for mode-matching and two mirrors (M1, M2) were used for laser-cavity alignment. The electrical part of the system consists of a laser controller, an electro-optic modulator (EOM) and its driver, a PID controller, a PZT driver, two near-infrared photodetectors, a data acquisition (DAQ) card and a laptop computer. The laser controller (model D2-105-500, Vescent photonics, Arvada, CO, USA), consists of a current driver and a temperature controller, capable of ultra-low current noise suitable for precision spectroscopic applications. An EOM, placed between the diode laser and the cavity, was used to modulate the laser frequency with a modulation frequency of 25 MHz. The reflected light signal from the cavity was directed to a photodetector (PD-AC200-InGaAs, QUBIG, Munich, Germany) and then sent to the EOM driver (model 1~200 MHz EOM Driver, QUBIG, Munich, Germany). Then, the reflected signal was mixed with the local oscillator signal after a phase shift and filtered to generate an error signal. The error signal was filtered by a low-pass filter with a 3-dB cutoff frequency of 1.9 MHz and amplified to control the diode laser output. A PID controller (PID110, TOPTICA, Munich, Germany) was used to generate a feedback signal to lock the laser to the cavity. The transmitted light from the cavity was focused by a lens L3 and was detected by a photodiode detector (PD2, PD-AC200-InGaAs, Thorlabs, Newton, NJ, USA). The signal was sent to a laptop via a DAQ card for data acquisition and analysis. A three-channel PZT controller (MDT693, Thorlabs, Newton, NJ, USA) was used to adjust the cavity length. A reference Laser Direct Absorption Spectroscopy (LDAS)-based detection channel, which consisted of a 1.36 µm reference diode laser (1E5GAAA, NEL, Yokohama, Japan), a multi-pass gas cell with a 3.7 m effective optical path (Sentinel Photonics, Princeton, NJ, USA) and a photodiode detector (PD3, PDA10CS, Thorlabs) were used to determine the H_2_O concentration in the cavity cell for calibration. The multi-pass gas cell was connected to the cavity cell by a gas tube to acquire the targeted trace gas species. Details of the LDAS technique are described in Ref. [28]. 

### 2.2. Fabry-Perot Cavity

**Figure 2:**
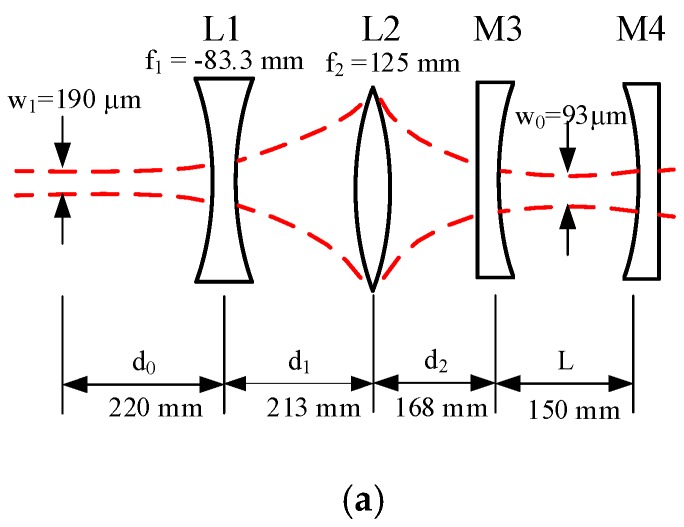

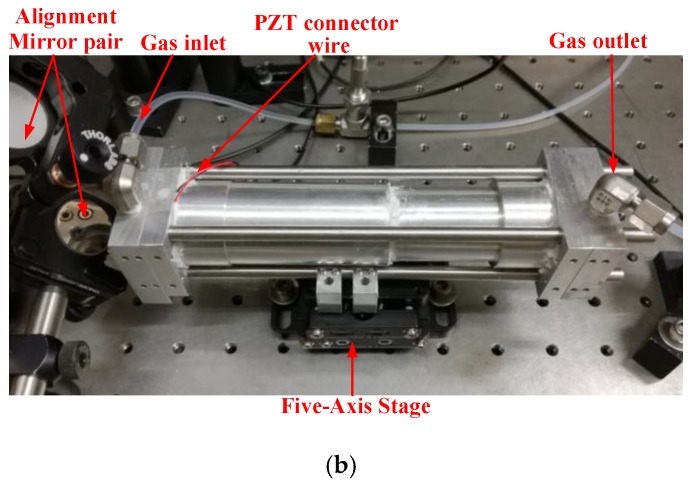
(**a**) Mode matching design of the cavity. (**b**) Photograph of the sealed F-P cavity with gas inlet and outlet connectors.

Mode matching design of the cavity is shown in Figure 2a. The focal lengths of the concave lens L1, convex lens L2, lens spacing distances *d*_0_, *d_1_*, *d_2_* were chosen to obtain a beam width and curvature to match the modes of the F-P cavity, which consist of two concave mirrors with a ROC (radius of curvature) *R*. The diode laser beam radius has a minimum at the center of the cavity and the beam waist radius is equal to the cavity waist radius. Hence, the incident diode laser beam must be focused on this point. The waist radius of the incoming diode laser beam is given by:

(1) $$w_{0}=\sqrt{k\sqrt{L(2R-L)}}$$

where *L* is the distance between two cavity mirrors and *k* = 2π/*λ* is the wave vector. For a Gaussian laser beam input, we consider the waist radius to be *w*_1_ and the distance to L1 to be *d*_0_. Based on the ABCD formalism [29], after L1 the waist radius of the exiting beam becomes *w*_2_ and the distance to L1 becomes *d*_1_, which can be determined using Equations (2) and (3):

(2) $$w_{2}=\sqrt{\frac{f_{1}{}^{2}w_{1}{}^{2}}{{\left(f_{1}-d_{0}\right)}^{2}+{\left(\frac{1}{2}kw_{1}{}^{2}\right)}^{2}}}$$

(3) $$d_{1}=f_{1}+\frac{(d_{0}-f_{1})f_{1}{}^{2}}{{\left(d_{0}-f_{1}\right)}^{2}+{\left(\frac{1}{2}kw_{1}{}^{2}\right)}^{2}}$$

After L2, the waist radius of the exiting beam becomes *w*_3_ and the distance to L1 becomes *d*_2_. Based on the ABCD formalism we obtain:

(4) $$w_{3}=\sqrt{\frac{f_{2}{}^{2}w_{2}{}^{2}}{{\left(f_{2}-d_{1}\right)}^{2}+{\left(\frac{1}{2}kw_{2}{}^{2}\right)}^{2}}}$$

(5) $$d_{2}=f_{2}+\frac{(d_{1}-f_{2})f_{2}{}^{2}}{{\left(d_{1}-f_{2}\right)}^{2}+{\left(\frac{1}{2}kw_{2}{}^{2}\right)}^{2}}$$

After M3 the waist radius *w*_3_ should be equal to *w*_0_, the distance to M3 becomes d_3_. Therefore, based on ABCD formula we obtain:

(6) $$d_{3}=L/2=f_{3}+\frac{w_{0}}{w_{2}}\sqrt{f_{3}{}^{2}-{\left(\frac{kw_{0}w_{2}}{2}\right)}^{2}}$$

The final parameters of the cavity and matched optics were calculated and are shown in Figure 2a. 

A further limitation of cavity length is due to optical stability. The relevant condition for a linear cavity with mirrors having radii of curvature *r*_1_ and *r*_2_ spaced by length *L* is:

(7) $$0<(1-\frac{L}{r_{1}})(1-\frac{L}{r_{2}})<1$$

A photograph of the sealed F-P cavity is shown in Figure 2b. The cavity is composed of an aluminum tube and two dielectric mirrors (EKSMA optics, Vilnius, Lithuania) with 100 mm radius of curvature and a 99.4% reflectivity at 1.53 μm. The thickness of the mirror is 3 mm. The two mirrors are fixed in a stainless steel cavity mount. The input cavity mirror is PZT-mounted in order to modulate the cavity length. Four stainless steel assembly rods were used to ensure cavity stability. The cavity was mounted to a five-axis stage by four pillars and the F-P system was assembled on an optical platform. The two ZnSe windows were used to seal the F-P cavity. The F-P cavity also acts as a gas cell, fitted with gas inlet and outlet connectors. The length of the cavity is 15 cm resulting in a free spectral range (FSR) of 0.9993 GHz, a linewidth of 1.92 MHz, a finesse of 522 and an effective absorption length of 49.7 m. 

### 2.3. Tuning Characteristics of the Laser

**Figure 3:**
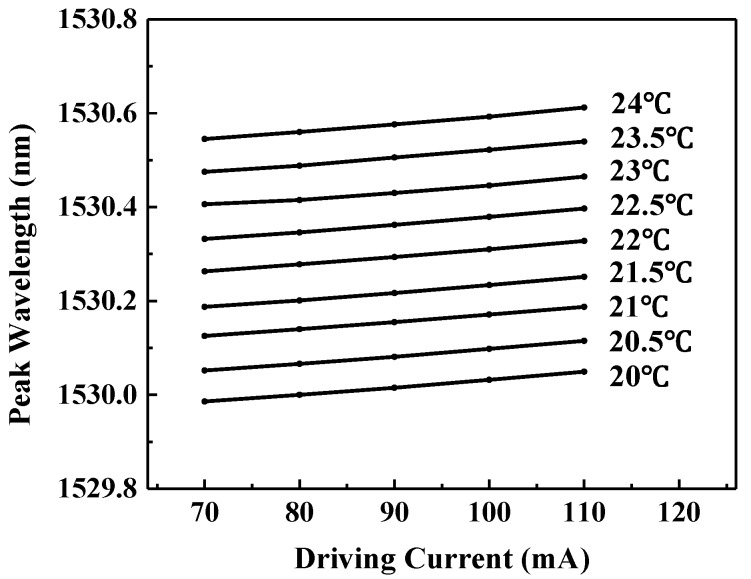
Tuning characteristics of the 1.53 µm DFB diode laser.

The wavelength tuning characteristics of the diode laser dependence on temperature and current were measured using the precision wavelength meter as shown in Figure 3. The driving current was varied from 70 mA to 110 mA and the laser’s operating temperature was changed from 20 °C to 24 °C. Each point in Figure 3 was measured for a period of 10 min with a sampling period of 1 s. The wavelength fluctuation range is 8 × 10^–4^ nm for each data point. The current tuning coefficient is 0.015 nm/10 mA and the temperature tuning coefficient is 0.14 nm/°C. 

## 3. Experiments and Results

### 3.1. Cavity Transmission Spectrum

**Figure 4:**
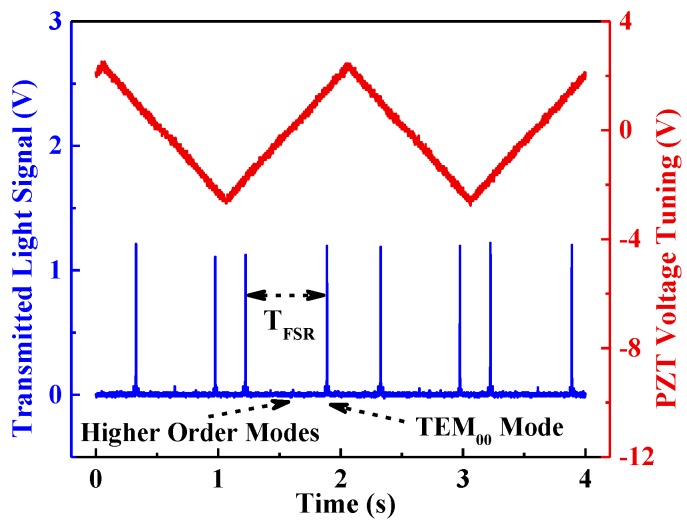
The cavity transmission spectrum. The red line shows the PZT driver voltage signal; the blue line shows the transmitted light signal changes following the applied PZT driver signal.

A 0.5 Hz ramp signal was applied to the PZT driver in order to sweep over a full cavity free-spectral range of ~1 GHz. The transmitted signal was viewed on an oscilloscope (MDO4104B-6, Tektronix, Beaverton, OR, USA), as shown in Figure 4. The fundamental mode (TEM_00_ mode) as well as higher order modes in the spectrum can be seen.

**Figure 5:**
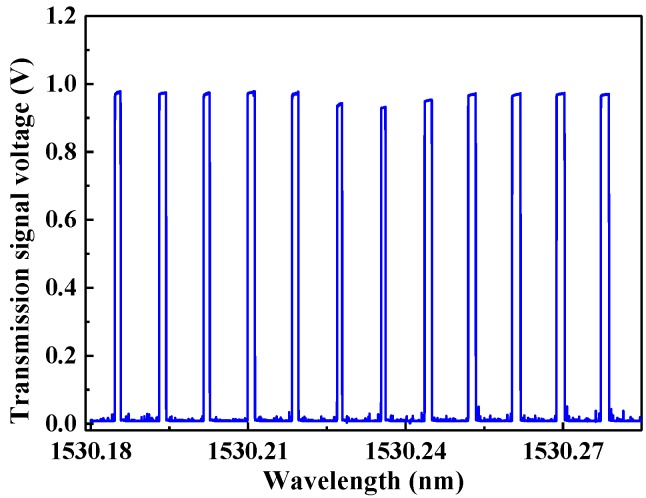
The PDH-locked cavity transmission signal of 1.6% H_2_O at 0.92 atm and 296 K. The target gas is air with a 1.6% H_2_O concentration as determined by the LDAS technique.

In order to enable the diode laser wavelength to sweep across a gas absorption line, the laser’s drive current was set to 89 mA, and the operation temperature was varied linearly from 21.4 °C to 22.1 °C at a frequency of 0.02 Hz. The emission peak wavelength changed from 1530.2 nm to 1530.283 nm and 12 FSRs were included in this range. Simultaneously, the PDH error signal passes through the PID controller and a voltage signal was generated to modulate the diode laser current. A LabVIEW-based trigger was designed to control the locking procedure. When the voltage signal amplitude exceeds a minimum threshold voltage, the PID controller starts to operate. The PID controller stops, when the voltage signal amplitude exceeds the maximum threshold voltage. In order to obtain the gas absorption spectrum, the diode laser must be repetitively locked to the cavity fundamental modes. Twelve mode locking signals generated in one period are shown in Figure 5. The important control parameter for the system is the PDH locking time, which can be changed by selecting the PID parameters. In this system, the locking time is 0.6 s, before the diode laser unlocks and waits to be relocked to the next cavity mode. In this manner, an absorption spectrum of H_2_O is obtained every 50 s.

### 3.2. Locked Wavelength with PZT Bias

**Figure 6:**
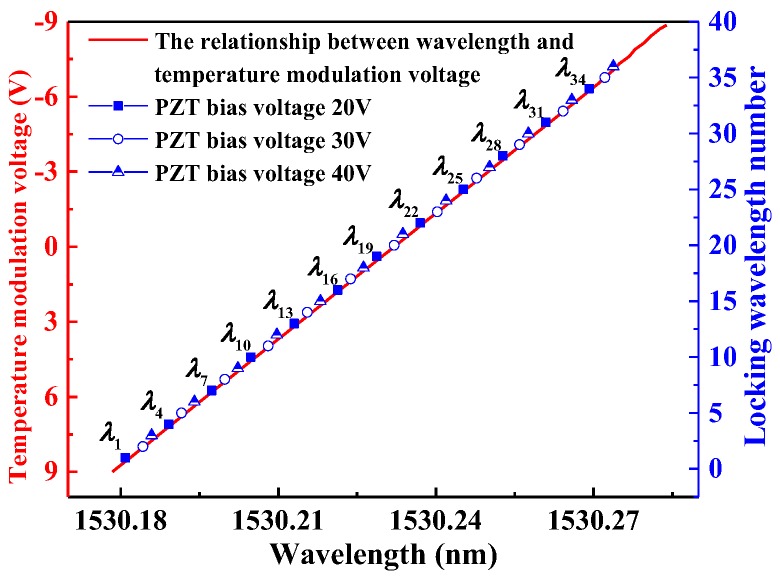
The acquired locking points in 3 scan periods with different PZT bias voltages of 20 V, 30 V and 40 V.

In Figure 6, the red line shows the relationship between wavelength and temperature modulation voltage. When the temperature modulation voltage is set to −9 V to 9 V, resulting in a temperature scan from 21.4 °C to 22.1 °C, 12 locking points are obtained (λ_1_, λ_4_, λ_7_, …, λ_34_) when the PZT bias voltage (i.e., the voltage applied to the PZT) is set to 20 V. Another 12 locking points (λ_2_, λ_5_, λ_8_, …, λ_35_) were obtained when the PZT bias voltage was changed to 30 V, and (λ_3_, λ_6_, λ_9_, …, λ_36_) were obtained when the PZT bias voltage was changed to 40 V. If these points are arranged in the order of wavelength, i.e., λ_1_, λ_2_, λ_3_, …, λ_36_, we can get an entire gas absorption line consisting of 36 locking points to represent the gas absorption.

**Figure 7:**
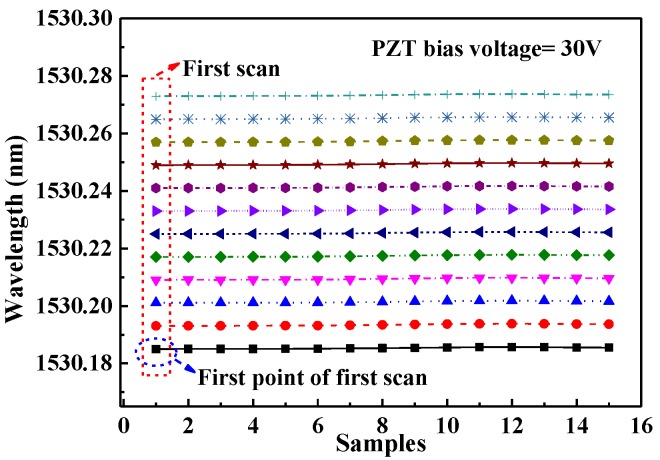
12 recorded mid-wavelengths at each locking period for 15 measurements, where the PZT bias voltage was 30 V.

In order to test the stability of the locking frequency in a scan period, 15 scans were acquired, and the mid-frequency of each locking point was recorded as shown in Figure 7. The floating ranges of the 15 scans are all less than 0.6 × 10^–3^ nm, indicating a good locking stability of the sensor cavity with the diode laser.

### 3.3. H_2_O Detection

#### 3.3.1. H_2_O Absorption Spectroscopy in the Near-Infrared

**Figure 8:**
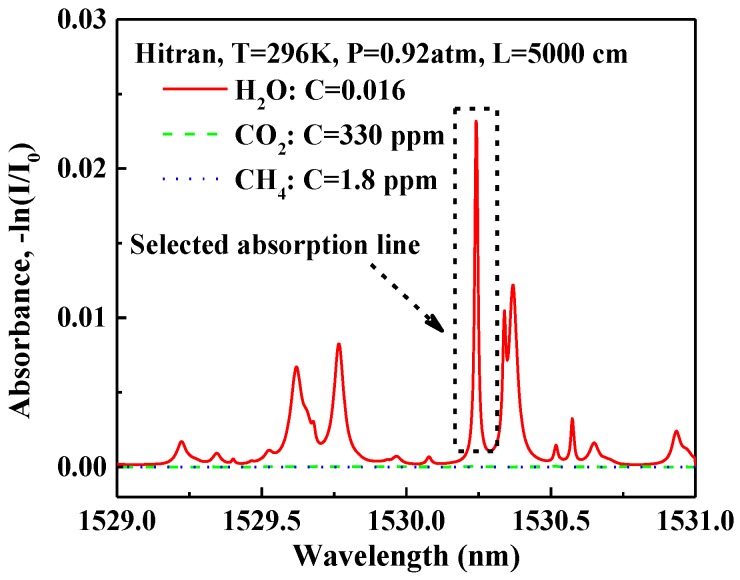
HITRAN-based absorption spectra of H_2_O in a spectral range from 1529 to 1531 nm at a pressure of 0.92 atm and a 50 m absorption length. (C: Concentration).

The H_2_O absorption spectrum based on the HITRAN 20212 [30] near 1.53 µm is depicted in Figure 8. The absorbance of H_2_O is 0.025 for a 50 m absorption length and a 1.6% H_2_O concentration. In order to examine the selectivity of this system and to avoid the effect of other gases in the atmosphere, an absorption spectrum of a typical atmospheric gas mixture (1.8 ppm CH_4_, 330 ppm CO_2_) is simulated as shown in Figure 8. In this range, other gases in the atmosphere have little interference.

#### 3.3.2. Experimental Results

**Figure 9:**
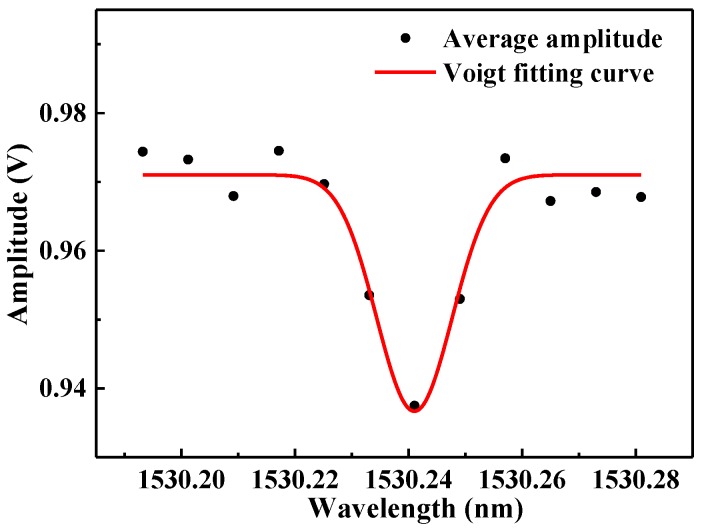
Ten averaged H_2_O absorption spectra at a concentration level of 1.6%, a pressure of 0.92 atm and a temperature of 296 K.

In order to test the effectiveness of the system, H_2_O detection was performed. The environmental temperature was 23 °C, and the pressure was controlled to be 0.92 atm. The driver current of the diode laser was set to 89 mA and the operating temperature was changed from 21.3 °C to 22.1 °C at a frequency of 0.02 Hz. In one period as the temperature rises, the diode laser will lock to the cavity 12 times for 1s intervals. 100 data points were recorded by a data-recording-and data-analyzing program based on the National Instruments LabVIEW software platform. In order to reduce fluctuations, the average value of the recorded 100 data points was calculated. The red line is the Voigt fitting curve of the H_2_O absorption spectrum. The maximum absorption signal (amplitude difference between the baseline and the absorption dip) is 0.033 V at a 1.6% H_2_O concentration determined by the reference LDAS-based sensor system, as shown in Figure 9. 

**Figure 10:**
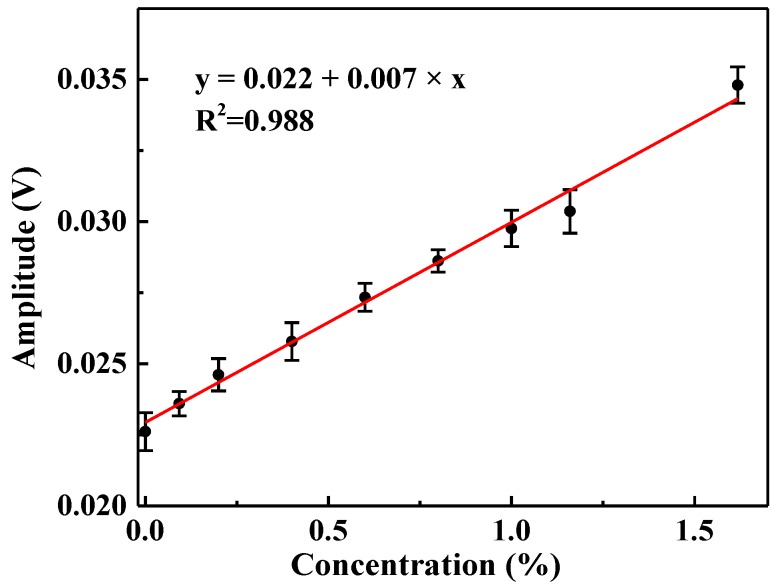
The fitting curve of the relationship between the absorption signal amplitude and H_2_O concentration within the range of 0–1.6%.

H_2_O mixtures with different concentration levels were obtained by passing pure N_2_ through a humidifier. The actual H_2_O concentration was determined by using the multi-pass cell-based LDAS system (shown in Figure 1). Measurements at different H_2_O concentrations were conducted. The amplitude of the absorption dip was recorded and the relationship between amplitude and H_2_O concentration was measured by reference detection channel and is shown in Figure 10. For each concentration, the amplitude is the average value of 10 measurement results. The obtained fitting equation between H_2_O concentration and the amplitude is *y* = 0.022 + 0.007 × *x*. The measured amplitude is linear with respect to H_2_O concentration with a linearity-dependent coefficient value of *R*^2^ = 0.988. 

**Figure 11:**
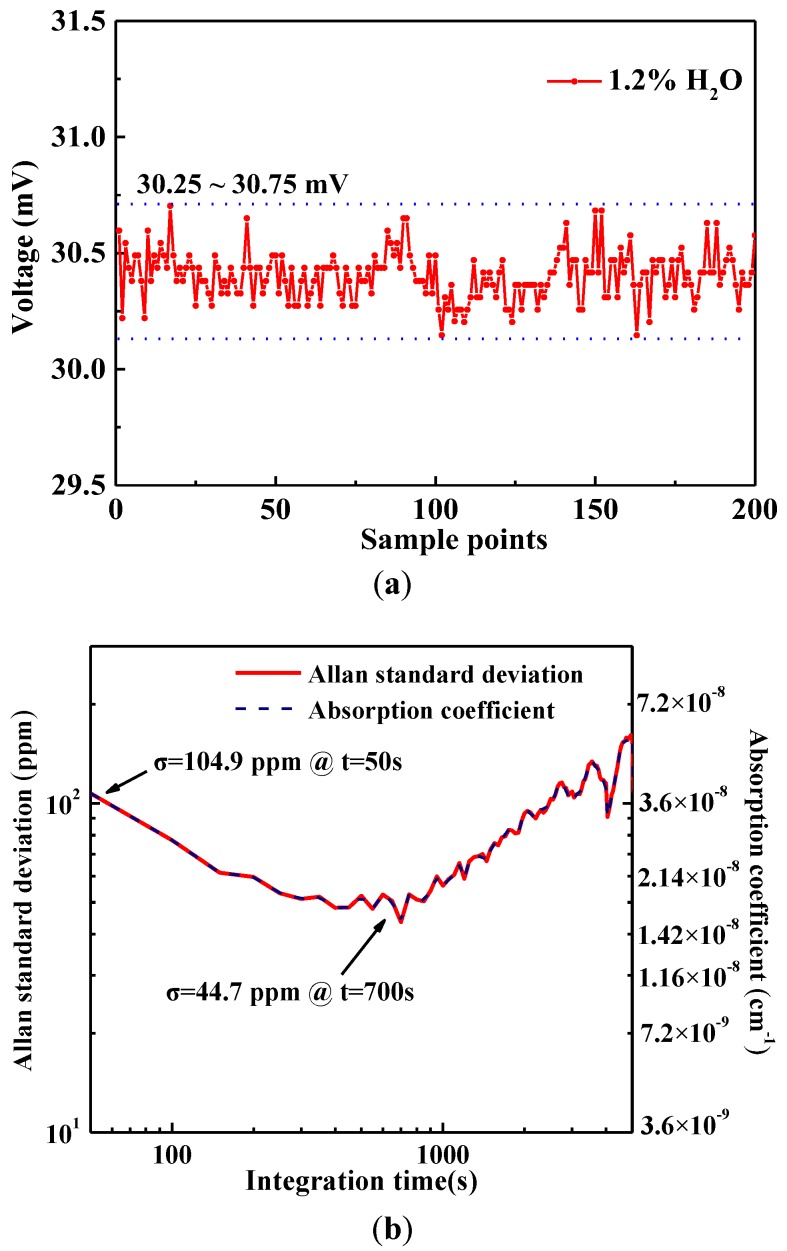
(**a**) Long-term monitoring of a standard 1.2% H_2_O sample. (**b**) Allan variance plot of the sensor system for the 1.2% H_2_O measurements.

In order to test the system’s stability, a H_2_O sample with a concentration level of 1.2% was measured over a period of 3 h with a sampling period of 50 s (shown in Figure 11a). The transmitted signal voltage varies in the range of 30.25~30.75 mV, indicating a H_2_O concentration variation range of 1.179~1.25%. An Allan standard deviation analysis was performed in order to determine the limit of detection (LOD) of this system as shown in Figure 11b. A detection sensitivity of 104.9 ppm is obtained based on an Allan standard deviation analysis for an averaging time of 50 s. When the averaging time increases to 700 s, the LOD reduces to 44.7 ppm by removing the white noise and the 1/f noise and the measured minimum detectable absorption coefficient is 1.5 × 10^–8^ cm^–1^. After 700 s, the Allan standard deviation increases due to etalon effects of the optical components.

## 4. Discussion and Conclusions

Simultaneous H_2_O measurements were performed using the CEAS sensor system and a reference LDAS sensor system. The measurements lasted for 85 min, 70 min, and 43 min for 3 concentration levels of ~1.6%, ~1.2% and ~0.09%. The measurement results of the two techniques are 1.64 ± 0.045% and 1.61 ± 0.008% (1 σ) for the first H_2_O sample, 1.124 ± 0.044% and 1.195 ± 0.0036% (1 σ) for the second H_2_O sample, and 0.121 ± 0.0351% and 0.094 ± 0.0028% (1 σ) for the third H_2_O sample. For the three cases, the ratios between the standard deviations were 5.6:1, 12.2:1, and 12.5:1, respectively. Based on the HITRAN 2012 database, the absorption coefficient for LDAS (3.7 m, 1.36 µm, 1%) is 1.75 and the absorption coefficient for CEAS (50 m, 1.53 µm, 1%) is 0.011, leading to a ratio is 159:1. This value is larger than the three ratios of 5.6:1, 12.2:1, and 12.5:1, which indicates that the signal-to-noise ratio of the CEAS system is larger than the LDAS system. Compared to LDAS, the reported sensor system reached a 49.7 m effective optical path length by a compact F-P cavity. The effective path length can be further improved by increasing the cavity length and the reflectivity of the cavity mirrors. The frequency stability of the diode laser was improved with a PDH locking technique, which reduced the noise level of the sensor. Compared to other CEAS-based system, a large laser wavelength tuning range was obtained using the RML-CEAS technique by temperature scanning (0.14 nm/°C), which can sweep across multiple gas absorption lines. Hence this technique can be applied to the simultaneous detection of multiple gases.

In conclusion, a PDH-locked repetitively mode-locked cavity-enhanced sensor system in near-infrared was developed and H_2_O detection measurements were conducted to demonstrate its performance of gas detection. A DFB diode laser with a wavelength of ~1.53 µm was employed as the light source and a PDH technique was used to lock the diode laser to a 15 cm resonant cavity. The FSR of the cavity is 1 GHz, and the effective optical path length is 49.7 m. H_2_O measurements were performed with this sensor system to demonstrate its gas sensing performance. A H_2_O absorption spectrum near 1.53 µm with good linearity between absorption spectrum amplitude and the H_2_O concentration was obtained. The LOD is ~104.9 ppm for a 50 s averaging time based on an Allan variance analysis and can be further improved to 44.7 ppm for an averaging time of 700 s, which represents a minimum detectable absorption coefficient of 1.5 × 10^–8^ cm^–1^. This sensor system has the potential for applications in sensitive trace gas detection.

## References

[1] Hamilton D.J., Orr-Ewing A.J. (2011). A quantum cascade laser-based optical feedback cavity-enhanced absorption spectrometer for the simultaneous measurement of CH_4_ and N_2_O in air. Appl. Phys. B.

[2] Dong L., Li C., Sanchez N.P., Gluszek A.K., Griffin R.J., Tittel F.K. (2016). Compact CH_4_ sensor system based on a continuous-wave, low power consumption, room temperature interband cascade laser. Appl. Phys. Lett..

[3] Yu Y., Sanchez N.P., Griffin R.J., Tittel F.K. (2016). CW EC-QCL-based sensor for simultaneous detection of H_2_O, HDO, N_2_O and CH_4_ using multi-pass absorption spectroscopy. Opt. Express.

[4] Richard L., Ventrillard I., Chau G., Jaulin K., Kerstel E., Romanini D. (2016). Optical-feedback cavity-enhanced absorption spectroscopy with an interband cascade laser: Application to SO_2_ trace analysis. Appl. Phys. B.

[5] Gianella M., Ritchie G.A.D. (2015). Cavity-Enhanced Near-Infrared Laser Absorption Spectrometer for the Measurement of Acetonitrile in Breath. Anal. Chem..

[6] Morville J., Kassi S., Chenevier M., Romanini D. (2005). Fast, low-noise, mode-by-mode, cavity-enhanced absorption spectroscopy by diode-laser self-locking. Appl. Phys. B.

[7] Romanini D., Chenevier M., Kassi S., Schmidt M., Valant C., Ramonet M., Jost H.J. (2006). Optical–feedback cavity–enhanced absorption: A compact spectrometer for real–time measurement of atmospheric methane. Appl. Phys. B.

[8] Kowzan G., Lee K.F., Paradowska M., Borkowski M., Ablewski P., Wójtewicz S., Masłowski P. (2016). Self-referenced, accurate and sensitive optical frequency comb spectroscopy with a virtually imaged phased array spectrometer. Opt. Lett..

[9] Abe M., Iwakuni K., Okubo S., Sasada H. (2014). Design of cavity-enhanced absorption cell for reducing transit-time broadening. Opt. Lett..

[10] Nation M., Wang S., Goldedstein C.S., Sun K., Davidson D.F., Jeffries J.B., Hanson R.K. (2015). Shock-tube measurements of exciter oxygen atoms using cavity-enhanced absorption spectroscopy. Appl. Opt..

[11] Boyson T.K., Dagdigian P.J., Pavey K.D., FitzGerald N.J., Spence T.G., Moore D.S., Harb C.C. (2015). Real-time multiplexed digital cavity-enhanced spectroscopy. Opt. Lett..

[12] Baran S.G., Hancock G., Peverall R., Ritchie G.A., Leeuwen N.J.V. (2009). Optical feedback cavity enhanced absorption spectroscopy with diode lasers. Analyst.

[13] Johnston P.S., Lehmann K.K. (2008). Cavity enhanced absorption spectroscopy using a broadband prism cavity and a supercontinuum source. Opt. Express.

[14] Yi H., Wu T., Wang G., Zhao W., Fertein E., Coeur C., Chen W. (2016). Sensing atmospheric reactive species using light emitting diode by incoherent broadband cavity enhanced absorption spectroscopy. Opt. Express.

[15] Zimmermann H., Ropcke J., Helden J.H.V., Wiese M., Lang N., Macherius U. (2016). Sensitive CH_4_ detection applying quantum cascade laser based optical feedback cavity-enhanced absorption spectroscopy. Opt. Express.

[16] Gianella M., Reuter S., Aguila A.L., Ritchie G.A.D., van Helden J.P.H. (2016). Detection of HO_2_ in an atmospheric pressure plasma jet using optical feedback cavity-enhanced absorption spectroscopy. New J. Phys..

[17] Kassi S., Campargue A., Mondelain D., Tran H. (2015). High pressure Cavity Ring Down Spectroscopy: Application to the absorption continuum of CO_2_ near 1.7 µm. Quant. Spectrosc. Radiat. Transfer.

[18] Zhao W., Xu X., Fang B., Zhang Q., Qian X., Wang S., Liu P., Zhang W., Wang Z., Liu D., Huang Y. (2017). Development of an incoherent broad-band cavity-enhanced aerosol extinction spectrometer and its application to measurement of aerosol optical hygroscopicity. Appl. Opt..

[19] Gherman T., Venables D.S., Vaughan S., Orphal J., Ruth A.A. (2007). Incoherent broadband cavity-enhanced absorption spectroscopy in the near-ultraviolet: Application to HONO and NO_2_. Environ. Sci. Technol..

[20] Kasyutich V.L., Canosa-Mas C.E., Pfrang C., Vaughan S., Wayne R.P. (2002). Off-axis continuous-wave cavity-enhanced absorption spectroscopy of narrow-band and broadband absorbers using red diode lasers. Appl. Phys. B.

[21] Leen J.B., Yu X.Y., Gupta M., Baer D.S., Hubbe J.M., Kluzek C.D., Tomlinson J.M., Hubbell M.R. (2013). Fast in situ airborne measurement of ammonia using a mid-infrared off-axis ICOS spectrometer. Environ. Sci. Technol..

[22] Centeno R., Mandon J., Cristescu S.M., Axner O., Harren F.J.M. (2015). External cavity diode laser-based detection of trace gases with NICE-OHMS using current modulation. Opt. Express.

[23] Foltynowicz A., Schmidt F.M., Ma W., Axner O. (2008). Noise-immune cavity-enhanced optical heterodyne molecular spectroscopy: Current status and future potential. Appl. Phys. B.

[24] Black E.D. (2001). An introduction to Pound–Drever–Hall laser frequency stabilization. Am. J. Phys..

[25] Langridge J.M., Laurila T., Watt R. S., Jones R.L., Kaminski C.F., Hult J. (2008). Cavity enhanced absorption spectroscopy of multiple trace gas species using a supercontinuum radiation source. Opt. Express.

[26] Cygan A., Lisak D., Masłowski P., Bielska K., Wojtewicz S., Domyslawska J., Trawinski R.S., Ciurylo R., Abe H., Hodges J.T. (2011). Pound-Drever-Hall-locked, frequency-stabilized cavity ring-down spectrometer. Rev. Sci. Instrum..

[27] Drever R., Hall J.L., Kowalski F., Hough J., Ford G., Munley A., Ward H. (1983). Laser phase and frequency stabilization using an optical resonator. Appl. Phys. B.

[28] Zheng C., Ye W., Sanchez N.P., Li C., Dong L., Wang Y., Griffin R. J., Tittel F.K. (2017). Development and field deployment of a mid-infrared methane sensor without pressure control using interband cascade laser absorption spectroscopy. Sens. Actuators B Chem..

[29] Kogelnik H., Li T. (1966). Laser beams and resonators. Proc. IEEE.

[30] Rothman L.S., Gordon I.E., Babikov Y., Barbe A., Benner D.C., Bernath P.F., Birk M., Bizzocchi L., Boudon V., Brown L.R. (2013). The HITRAN2012 molecular spectroscopic database. J. Quant. Spectrosc. Radiat. Transf..

